# Antibacterial Effect of Juglans Regia Bark against Oral Pathologic Bacteria

**DOI:** 10.1155/2013/854765

**Published:** 2013-07-07

**Authors:** Faramarz Zakavi, Leila Golpasand Hagh, Arash Daraeighadikolaei, Ahmad Farajzadeh Sheikh, Arsham Daraeighadikolaei, Zahra Leilavi Shooshtari

**Affiliations:** ^1^Department of Operative and Esthetic Dentistry, School of Dentistry, Ahvaz Jundishapour University of Medical Sciences, Ahvaz 613571-5775, Iran; ^2^Department of Periodontology, School of Dentistry, Ahvaz Jundishapour University of Medical Sciences, Ahvaz 613571-5775, Iran; ^3^Ahvaz Dental School Research Center, Ahvaz 613571-5775, Iran; ^4^Department of Microbiology, School of Medicine, Ahvaz Jundishapur University of Medical Sciences, Ahvaz 61355-45, Iran; ^5^Department of Pharmacognosy Clinical, Kerman School of Pharmacy, Kerman 76135-1614, Iran

## Abstract

*Background*. In this study antimicrobial effect of ethanolic and aqueous extracts of Juglans regia bark in Iran was evaluated on four different oral bacteria, *Streptococcus mutans*, *Streptococcus salivarius*, *Streptococcus sanguis*, and *Staphylococcus aureus*. *Methods*. Aqueous and ethanol extracts of Juglans regia bark were prepared by using disk diffusion technique and Minimal Inhibitory Concentration (MIC) methods. Tetracycline 30 **μ**g and Erythromycin 15 **μ**g were used as positive control and water as negative control in disk diffusion and MIC methods. Data were analyzed by ANOVA test. *Results*. The results showed that *S. sanguis* and *S. mutans* were the most sensitive and the most resistant bacteria against ethanolic and aqueous extracts, respectively. Ethanolic extract had significant antibacterial effect against all tested bacteria. Aqueous extract did not show antibacterial effect on *S. mutans*, in contrast to ethanolic extract. Aqueous extract had significantly antibacterial effect against *Staphylococcus aureus*, *S. salivarius*, and *S. sanguis* compared to control (*P* < 0.0001), but it did not show effect on *S. mutans* when compared with Erythromycin. According to the obtained MIC values, ethanol extract of Juglans regia bark had the lowest rate. *Conclusion*. The results may provide the basis for using natural antimicrobial substance for oral hygiene prophylaxis purposes.

## 1. Introduction

Oral cavity hygiene has gained significance in recent years. Antimicrobial agents are usually incorporated into hygiene products for the treatment and prevention of plaque and gingivitis [1]. Dental caries is a public oral health problem and an infectious-contagious disease that implies an imbalance of normal molecular interactions between the tooth surface/subsurface and the adjacent bacterial biofilm [2]. The majority of the population may not carry out mechanical plaque removal adequately. Thus, antimicrobial mouth rinses that augment daily home care may provide an efficient income of remove or controlling bacterial plaque to limit gingivitis and periodontitis [3]. Here is a continuous need of new antimicrobial components due to rapid appearance of multiple drug-resistance bacteria [4]. Plants drugs are known to have protection systems beside pathogenic bacteria [5]. The genus juglans (family Juglandaceae) comprises several species and is widely dispersed throughout the world. Many parts of Green walnuts such as shells, kernel and seed, bark, and leaves are used in the pharmaceutical and beauty industry [6, 7]. Juglans regia L. bark is used in some countries as a toothbrush and as a dye for coloring the lips for makeup purpose [8]. Walnut (Juglans regia L.) bark has been claimed to own anti-inflammatory, blood purify, anticancer, depurative, diuretic, and laxative activities. It contains several therapeutically active constituents, particularly polyphenols [9].

Juglans regia stem bark contains chemical constituents, namely, *β*-sitosterol, ascorbic acid5, juglone, folic acid, gallic acid, regiolone, and quercetin-3-*α*-L-arabinoside [10, 11]. Antifungal, antibacterial, and antioxidant activities of this plant have been described [12–16].

Its extract of Juglans regia bark showed a broad spectrum antimicrobial activity in a dose-dependent manner. It inhibited the growth of several pathogenic microorganisms such as Gram-positive bacteria (*Staphylococcus aureus* and *Streptococcus mutans*), Gram-negative bacteria (*Escherichia coli* and *Pseudomonas aeruginosa*), and pathogenic yeast (*Candida albicans*). The extract has either synergistic or additive act when tested with a broad variety of antibacterial drugs. Juglans regia also increased the pH of saliva [8]. Rahul tested the stem bark extracts of J. regia L. for antimicrobial activity against the microbes present in the saliva specimens of patients suffering from dental caries. Acetone extract was found to be more effective of the extracts as antimicrobial against the oral microflora [4].

Since many of currently used natural products are either detrimental or ineffective, natural folk medicines with antimicrobial effects have been under investigation during the few past decades. Growing emphasis of plant studies in the field of dentistry is due to the antibiotic-resistant bacteria, side effects of chemical antibiotics, and their high cost in developing countries. Antimicrobial activity of Walnut tree comes from its chemical composition. Juglans regia bark species have been analyzed chemically adequately in several studies, and in agreement among most of them, with respect to the essential oil composition, the major components are phenolic compounds, terpenoids, alkaloids, flavonoids, and steroids [17]. It is reported that leaves from J. regia L. contain monoterpenes and sesquiterpenes, and the bark contains ketones like juglone, regiolone, sterol, and flavonoid [18].

Juglans regia bark is a medicinal plant used in Iranian folk medicine as antimicrobial medicine [19, 20].

The aim of the present study was to evaluate the antimicrobial activities of ethanolic and aqueous extracts of Juglans regia bark against four species of oral bacteria.

## 2. Material and Methods

### 2.1. Collection and Identification of Plant Samples

The plant material (stem bark) of the species was collected from local market from kordestan, Iran, and confirmed at Department of Botany, School of Agriculture, Shahid Chamran University, Ahvaz, Iran. Samples were dried at room temperature and crushed in a grinder.

### 2.2. Preparation of the Aqueous and Ethanolic Extracts

Juglans regia bark powdered (100 g) was extracted by 500 mL of ethanol (for preparing the ethanolic extract) and 500 cc of water (for preparing the aqueous one) consecutively using Soxhlet extractor with not exceeding the boiling point of the solvent. The extracts were filtered by Whatman filter paper and then concentrated in vacuum at 40°C by means of a Rotary Evaporator. The residues obtained were stored in a freezer until future tests [21].

### 2.3. Determination of Antibacterial Activities

The bacteria strains were used for antibiogram pattern including *Streptococcus mutans* PTCC1683, *Streptococcus salivarius* PTCC1448, *Streptococcus sanguis* PTCC1449, and *Staphylococcus aureus* PTCC: 1112, were provided by the Iranian Microbial Type Culture Collection. The strains were inoculated in blood agar and incubated at 37°C at least for 24 h, until emergent adequate colonies. The bacteria strains were touching to 4-5 colonies raised from pure microorganism culture and inoculated at the concentration in order to attain the Mc. Farland No: 0.5 density and then *streptococci* species and *Staphylococcus aureus* incubated in Muller-Hinton and blood agar, respectively.

### 2.4. Disc-Diffusion Assay

Extracts were diluted in water, and thus 1 gr of each extract inoculated in 1 mL distil water and diluted it to obtain different concentrations as 1000, 500, 250, 125, and 62.5 mg/mL for aqueous and ethanolic extracts. The blank discs (Padtan Teb Co, Iran) were inoculated with 20 *μ*L of every concentration extracts and placed on the Muller-Hinton and blood agar were cultivated with bacterial strains. Negative controls used the same solvents to dissolve the extracts, and Tetracycline (30 *μ*g) and Erythromycin (15 *μ*g) were used as positive references. The inoculated plates were incubated at 37°C for 18 hours. Antimicrobial pattern was evaluated by measuring the zone of inhibition against the test bacteria based on millimeters.

### 2.5. Determination of Minimum Inhibitory Concentration (MIC) by *E* Test

The MIC values were read as the antibacterial concentration at the point where dense colonial growth intersected the disc [22, 23]. The assays were performed three times for each bacterium.

### 2.6. Statistical Analysis

Analysis of variance (ANOVA) was used to determine the significance (*P* ≤ 0.05) of the data obtained in all tests.

## 3. Results

The inhibition zones due to aqueous extract, negative control (water), and positive control (Tetracycline 30 *μ*g, Erythromycine 15 *μ*g) are showed in Table 1. Aqueous extract had significantly antibacterial effect against *Staphylococcus aureus*, *S. salivarius* (Figure 2), and *S. sanguis* (Figure 4) compared to control and was significant (*P* < 0.0001), but it did not show effect on *S. mutans* when compared with Erythromycin. Also the results were shown that ethanolic extracts were significant (*P* < 0.0001) with inhibitory effect on the growth of four tested bacteria, in contrast to negative control (Table 2).

**Figure 1:**
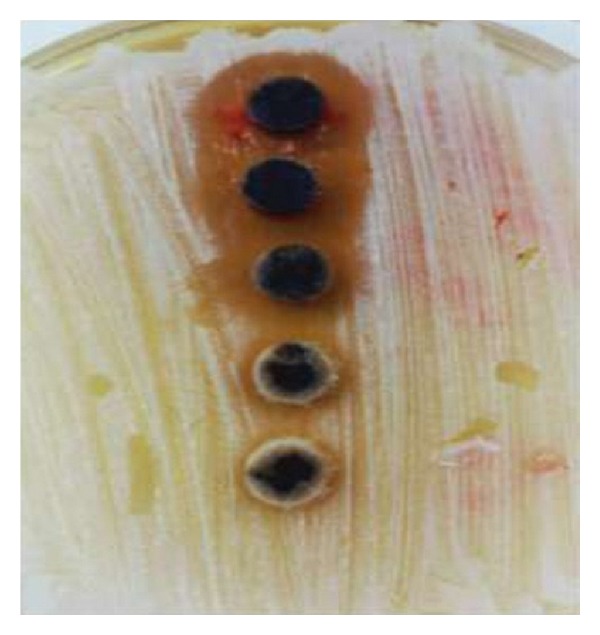
The effects of ethanolic extracts of juglans regia bark against *S. salivarius*.

**Figure 2:**
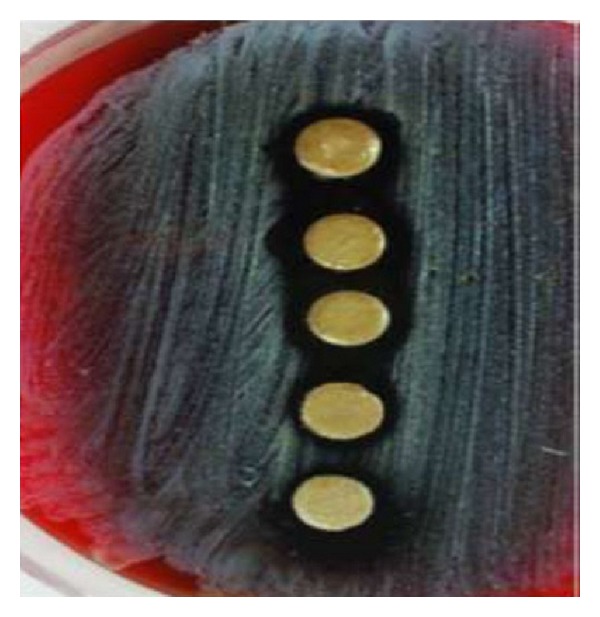
The effects of aqueous extracts of juglans regia bark against *S. salivarius*.

**Figure 3:**
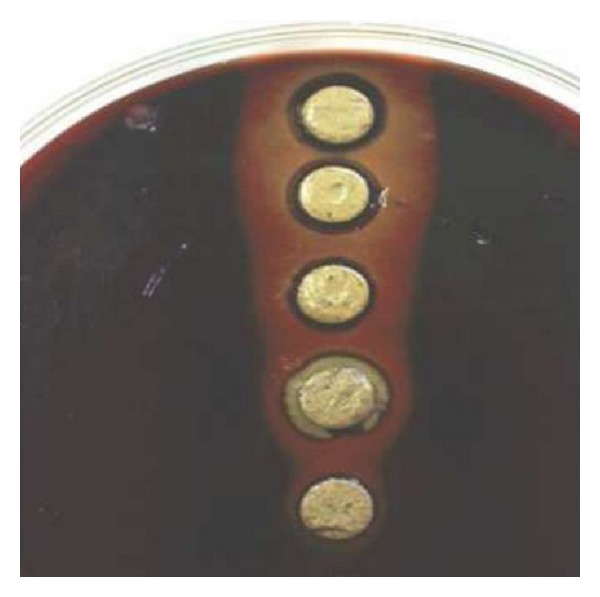
The effects of ethanolic extracts of Juglans regia bark against *S. sanguis*.

**Figure 4:**
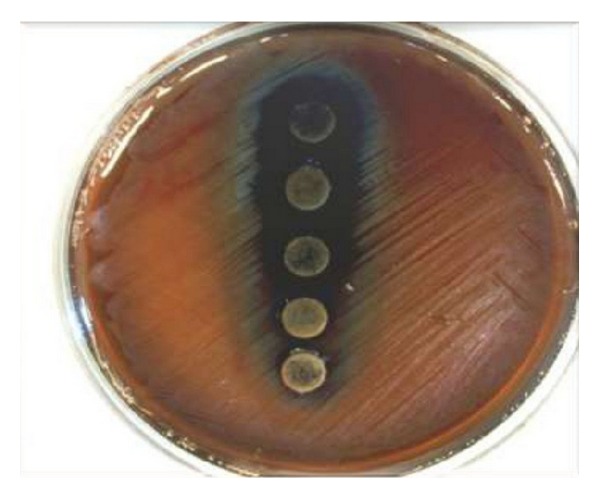
The effects of aqueous extracts of Juglans regia bark against *S. sanguis*.

The MIC was evaluated for the antimicrobial activity of ethanolic and aqueous extracts of Juglans regia on bacteria and the results were shown in Table 3. According to the obtained MIC values, ethanol extract of Juglans regia bark had the lowest MIC of 1.25 microg/mL on *S. sanguis* (Figure 3). Ethanol extract MIC value for *S. salivarius* (Figure 1) and Aqueous extract MIC value for *S. Sanguis* and *S. salivarius* were similar, 2.50 microg/mL. Antibacterial activity of the aqueous extract on *Staphylococcus aureus* was the least sensitivity with a highest determined MIC of 125 microg/mL, while MIC value for the ethanolic extract was 2.00 microg/mL.

Aqueous extract did not have any inhibitory effect on *S. mutans* in terms of antimicrobial activity; however, for the ethanol extract it was 5.00 microg/mL.

## 4. Discussion

The most important cause of gingival inflammation and dental caries is bacterial plaque. Some of people keep away from chemical mouth rinse because of the presence of alcohol, artificial preservation, or artificial color in mouth rinses [24]. So, recently, researches for using of medicinal plants are increasing. The present study showed that Juglans regia are potential antimicrobial agents and can be used in oral hygiene products. 

According to our finding, we indicated that the high concentrations of ethanolic and aqueous extracts have had antimicrobial effects against *S. sanguis*, *S. mutans*, *S. salivarius*, and *staphylococcus aureus* with significant difference in contrast to control. Also we showed that this effect was dose dependent. We indicated that ethanolic extract exhibited zones of inhibition against all the tested samples, whereas aqueous extract is active with comparatively smaller zones of inhibition (Tables 1 and 2). Deshpande et al. reported that the acetone extract of J. regia L was found to be more effective of the extracts as antimicrobial against the oral microflora [4]. Sharafati et al. showed that walnut leaves could be used as an easily available source of natural compounds to inhibit the growth of different Gram-positive bacteria responsible for dental plaques and oral hygiene problems [25]. Recently, Darmani et al. reported the growth inhibition of various cariogenic bacteria (*Streptococcus mutans*, *Streptococcus salivarius*, *Lactobacillus casei*, and *Actinomyces viscosus*) by walnut aqueous Extract [26].

The data of this study clearly indicated that ethanolic and aqueous extracts of juglans regia bark significantly inhibited the growth of the tested oral bacteria, and those reports are compatible with our finding. The antibacterial property of the plant material may be due to the presence of phenolic compounds, terpenoid, alkaloids, flavonoids, and steroids. The bark of juglone, regiolane, contains ketones, sterol, and flavonoid [10].

## 5. Conclusion

Many herbs have preventive or therapeutic potentials. This study has confirmed the antimicrobial potentials of this kind of Iranian plant, thus supporting its folklore application as a preventive remedy for various microbial diseases (caries and periodontal disease) in the oral cavity in Iran. It provides the basis for the present rapidly increasing interest for the use of natural antioxidants and antimicrobials. Further studies are required to find these effects in order to replace synthetic medications with natural remedies.

We concluded that Iranian bark of juglone, regiolane, has the antibacterial effects against the important oral bacteria, and ethanolic extract was of higher effectivity against tested bacteria than aqueous extract.

## Figures and Tables

**Table 1:** Average of inhibition zone of aqueous extract on some oral bacteria (mm).

Positive control (+)	Negative control (−)	Concentrations (mg/mL)					Microorganism	
		1000	500	250	125	62.5		
32	—	12	12	12	6.5	—	*Staphylococcus aureus*	Average zone of inhibition (mm)
17	—	25	15	10.5	—	—	*Streptococcus sanguis *	
26	—	13.5	11	9	—	—	*Streptococcus salivarius *	
20	—	—	—	—	—	—	*Streptococcus mutans *	

Negative control: water.

Positive control: Tetracycline (30 *μ*g) against *Streptococcus mutans*, *Streptococcus salivarius* and Erithromycin (15 *μ*g) against *Streptococcus sanguis*.

(—): no inhibition zone.

**Table 2:** Average of inhibition zone of ethanolic extract on bacteria (mm).

	Microorganism	Concentrations (mg/mL)					Negative control (−)	Positive control (+)
		1000	500	250	125	62.5		
Average zone of inhibition (mm)	*Staphylococcus aureus *	7	8	15	16	18		**32 **
	*Streptococcus sanguis *	—	8.75	11	12.5	14		**17 **
	*Streptococcus salivarius *	—	—	6.5	16	18		**26 **
	*Streptococcus mutans *	—	—	—	10	13		**20 **

Negative control: water.

Positive control: Tetracycline (30 *μ*g) against *Streptococcus mutans*, *Streptococcus salivarius* and Erithromycin (15 *μ*g) against *Streptococcus sanguis*.

(—): no inhibition zone.

**Table 3:** MIC values of etanolic and aqueous extracts of juglans regia bark against oral Bacteria.

Microorganism	PTCC	MIC (mg/mL)	
		Etanolic	Aqueous
*Staphylococcus aureus*	1112	2.00	125
*Streptococcus sanguis *	1449	1.25	2.50
*Streptococcus salivarius *	1448	2.50	2.50
*Streptococcus mutans*	1683	5.00	—

## References

[1] Şahin F, Karaman I, Güllüce M (2003). Evaluation of antimicrobial activities of *Satureja hortensis* L.. *Journal of Ethnopharmacology*.

[2] García-Cortés JO, Medina-Solís CE, Loyola-Rodriguez JP (2009). Dental caries’ experience, prevalence and severity in Mexican adolescents and young adults. *Revista de Salud Pública*.

[3] Barnett ML (2003). The role of therapeutic antimicrobial mouthrinses in clinical practice: control of supragingival plaque and gingivitis. *Journal of the American Dental Association*.

[4] Deshpande RR, Kale AA, Ruikar AD (2011). Antimicrobial activity of different extracts of *Juglans regia* L. against oral Microflora. *International Journal of Pharmacy and Pharmaceutical Sciences*.

[5] Smith E, Williamson E, Zloh M, Gibbons S (2005). Isopimaric acid from *Pinus nigra* shows activity against multidrug-resistant and EMRSA strains of *Staphylococcus aureus*. *Phytotherapy Research*.

[6] Pereira JA, Oliveira I, Sousa A (2007). Walnut (*Juglans regia* L.) leaves: phenolic compounds, antibacterial activity and antioxidant potential of different cultivars. *Food and Chemical Toxicology*.

[7] Stampar F, Solar A, Hudina M, Veberic R, Colaric M (2006). Traditional walnut liqueur—cocktail of phenolics. *Food Chemistry*.

[8] Alkhawajah AM (1997). Studies on the antimicrobial activity of *Juglans regia*. *American Journal of Chinese Medicine*.

[9] Bhatia K, Rahman S, Ali M, Raisuddin S (2006). *In vitro* antioxidant activity of *Juglans regia* L. bark extract and its protective effect on cyclophosphamide-induced urotoxicity in mice. *Redox Report*.

[10] Sharma PC, Yelne MB, Dennis TJ (2002). Bhunimba (Andrographis paniculata). *Data Base on Medicinal Plants Used in Ayurveda*.

[11] Joshi SG (2007). *Medicinal Plants*.

[12] Isanga J, Zhang GN (2007). Biological active components and nutraceuticals in peanuts and related products: review. *Food Reviews International*.

[13] Miraliakbari H, Shahidi F (2008). Oxidative stability of tree nut oils. *Journal of Agricultural and Food Chemistry*.

[14] Amaral JS, Casal S, Pereira JA, Seabra RM, Oliveira BPP (2003). Determination of sterol and fatty acid cmpositions, oxidative stability, and nutritional value of six walnut (*Juglans regia* L.) cultivars grown in Portugal. *Journal of Agricultural and Food Chemistry*.

[15] Noumi E, Snoussi M, Trabelsi N (2011). Antibacterial, anticandidal and antioxidant activities of *Salvadora persica* and *Juglans regia* L. extracts. *Journal of Medicinal Plant Research*.

[16] Noumi E, Snoussi M, Hajlaoui H, Valentin E, Bakhrouf A (2010). Antifungal properties of *Salvadora persica* and *Juglans regia* L. extracts against oral *Candida* strains. *European Journal of Clinical Microbiology and Infectious Diseases*.

[17] Bandow JE, Brotze H, Leichert LIO (2003). Proteomic approach to understanding antibiotic action. *Antimicrobial Agents and Chemotherapy*.

[18] Paulsena MT, Ljungman M (2005). The natural toxin juglone causes degradation of p53 and induces rapid H2AX phosphorylation and cell death in human fibroblasts. *Toxicology and Applied Pharmacology*.

[19] Mirheidar H (1995). *Knowledge of Plants*.

[20] Zargari A (1990). *Medical Plants*.

[21] Lin J, Opoku AR, Geheeb-Keller M (1999). Preliminary screening of some traditional Zulu medicinal plants for anti-inflammatory and anti-microbial activities. *Journal of Ethnopharmacology*.

[22] Amin M, Kapadnis BP (2005). Heat stable antimicrobial activity of Allium ascalonicum against bacteria and fungi. *Indian Journal of Experimental Biology*.

[23] Pfaller MA, Messer SA, Mills K, Bolmstrom A (2000). *In vitro* susceptibility testing of filamentous fungi: comparison of Etest and reference microdilution methods for determining itraconazole MICs. *Journal of Clinical Microbiology*.

[24] Haffajee AD, Yaskell T, Socransky SS (2008). Antimicrobial effectiveness of an herbal mouthrinse compared with an essential oil and a chlorhexidine mouthrinse. *Journal of the American Dental Association*.

[25] Sharafati-Chaleshtori R, Sharafati-Chaleshtori F, Rafieian-Kopaei M (2011). Biological characterization of Iranian walnut (*Juglans regia*) leaves. *Turkish Journal of Biology*.

[26] Darmani H, Nusayr T, Al-Hiyasat AS (2006). Effects of extracts of miswak and derum on proliferation of Balb/C 3T3 fibroblasts and viability of cariogenic bacteria. *International Journal of Dental Hygiene*.

